# Some Necessary Qualifications

**Published:** 1919-02-08

**Authors:** 


### THE POST OF MATRON:

### II. Some Necessary Qualifications. *(Concluded from Nov. 9, p. 117.)*

In a former article we considered some of the qualities which a good matron must possess. The subject of her qualifications is almost equally important. It seems hardly necessary, in these days, to say that a matron must have full training; but there may still be people who think that a heaven-born genius can manage without it. There were, in the old days, matrons, and good ones, who had only a year's training, but, first, much less knowledge was required of nurses; also, sisters and matrons were supposed to have a sort of intensive training, and were often a class apart—a state of things now happily obsolete. And, after all, they were often terribly handicapped by want of experience—for instance, by never having been on night duty, and by a lack of practical knowledge of theatre work.

It is a great advantage to a matron if, besides her general training, she has other certificates—midwifery, fever, massage. But even more important than these is the qualification of really good experience as a ward sister. It is most difficult for a matron to enter thoroughly into the sisters' point of view, to advise them about their work, and to arrange the staff in their wards, if she has not had much experience of charge duty herself. It would seem to be desirable, from this point of view, that a matron should have held all kinds of hospital posts, and also should have done private and district nursing. A wide professional experience is very helpful in training nurses, provided that too many years are not spent in acquiring it. A matron's work needs not only physical strength and energy, but the spring of hope and recovery from disappointment and trouble which few women keep when their youth is past. When "the clouds return after the rain" it is hard to start fresh schemes and new plans with the cheerful optimism that carries them to success.

Besides her training in strictly professional matters, it is very desirable that a matron should know something of business methods. If she is not naturally orderly and methodical this is all the more important. It is difficult to estimate the saving in time and temper effected by a matron whose papers and books are all in good order, her letters arranged for ready reference, all necessary information about staff and wards easily accessible. The saving is not only for the matron herself—her committee, doctors, and staff all share in it.

A knowledge of housekeeping on a large scale is another important qualification. Experience of this kind is already given to certificated nurses in some hospitals, by allowing them, in turn, to help the home sister. It would be an excellent thing if all nurses had some work of this kind. Grumbling would be diminished, and nurses would be much more sympathetic members of a household, if they knew something of the trials of housekeeping. It is most important that the staff of a hospital should be properly fed, and the home comfortable. But if the matron knows nothing of household matters, or is indifferent to them, the standard of comfort will go down, however good her subordinates may be.

In the same way, if the matron does not understand the care of linen the ward stock will soon be in a bad state. It is very hard for a ward sister to be careful and conscientious about this, if no one but herself takes an interest in it. But if "matron is so pleased to find the stock in good order," the dull subject becomes quite fairly interesting.

It is quite vain to try to look wise about matters in hospital that one really knows nothing about. Nurses either see through pretence of that kind, or say that they can't make matrons understand their difficulties.

In small hospitals the work of the theoretical teaching of probationers falls to the matron, at any rate as regards first-year nurses. In large schools, if there is no sister-tutor, this work is usually done by the assistant matron and home sister. Probably most matrons have held one of these posts, and have thus gained experience in teaching. They should have learned to teach methodically, by a syllabus, to arrange information clearly, to lecture in simple words, slowly and distinctly, and to make the various subjects interesting to their hearers. If probationers find lectures dull it is, in nine cases out of ten, the fault of the teacher.

In addition to necessary qualifications for her work, a matron should welcome every opportunity of widening her mind, of enlarging her experience, and of cultivating any talent she may possess. Knowledge of any kind, even apparently useless knowledge, is sure to be valuable to a matron at some time or other in her career. Nursing, in common with all other professions, tends to draw the worker's mind and heart into a narrow groove. The work is so absorbing that some nurses have no ideas beyond it, sometimes not even beyond the small part of it which is going on in their own hospital. It is a great help in counteracting this tendency in young nurses if their matron is a woman of cultivated mind, many interests, and broad sympathies. Would-be matrons should remember the advice of the wise man: "In a great matter or a small, be not ignorant."

